# The Association of *-475* and *-631* Interleukin-2 Gene Polymorphism with Multiple Sclerosis in Iranian Patients

**Published:** 2013-07-02

**Authors:** Aida Sayad, Abdolamir Allameh, Arezou Sayad, Mehrdad Noruzinia, Mohammad Taghi Akbari, Ali Sarzaeem, Akbari Akbar, Reza Haji Hoseini

**Affiliations:** 1 Department of Biochemistry, Payame Noor University, Tehran, Iran; 2 Department of Clinical Biochemistry, Faculty of Medical Sciences, Tarbiat Modares University, Tehran, Iran; 3 Departments of Medical Genetics, Shahid Beheshti University of Medical Sciences, Tehran, Iran; 4 Department of Medical Genetics, Faculty of Medical Science, Tarbiat Modares University, Tehran, Iran; 5 Department of Venomous Animals and Antivenom Production, Institute of Razi, Vaccine and Serum Research, Karaj, Iran; 6 Islamic Azad University, Tehran Olum and Tahghighat Branch, Tehran, Iran

**Keywords:** *interleukin-2 (IL2)*, Polymorphism, Multiple Sclerosis, Genetic Susceptibility

## Abstract

**Objective:**

Multiple sclerosis (MS) is a chronic autoimmune disease due to demyelination of the central nervous system. It is believed that cytokines are involved in the pathogenesis of MS. The *interleukin-2 (IL2)* gene is powerful functional candidate that is involved in immune regulation and operation. In this study, for the first time, we investigated the effect of *-475* A/T and *-631* G/A *IL2* polymorphisms on MS disease in Iranian patients.

**Materials and Methods:**

In this case-control study, 100 MS patients (mean age: 32.95 ± 6.51 years, age range: 20-42 years) selected according to McDonald criteria, and 100 ethnically, sex and age matched healthy controls (mean age: 29 ± 7.8 years, age range: 20-52 years) with no personal or family history of autoimmune diseases were studied. The restriction fragment length polymorphism-polymerase chain reaction (RFLP-PCR) method was applied to define different alleles and genotypes of *IL2* promoter single nucleotide polymorphism *-475* A/T as well as *-631* G/A among individuals. χ^2^ was calculated and Fisher’s exact test was applied to analyze the obtained data. The value of p < 0.05 was considered significantly .

**Results:**

Evaluation of the *-475 IL2* revealed that T allele and A/T genotype are present in 2% and 4% of MS patients, respectively, whereas T allele was absent in control samples. The comparison between alleles and genotypes in MS patients and healthy controls was not significant (p=0.1). For the *-631* position, 1% and 2% of MS patients carried A allele and A/G heterozygote genotypes, respectively. All control samples had G allele and G/G genotype. The differences between patients and controls were not significant (p=0.4). Moreover, our results showed a very low frequency of T at *-475* and A at *-631 IL2* position in each of the two groups.

**Conclusion:**

Both *-475* and *-631 IL2* polymorphisms were higher in MS patients as compared to controls, but the frequency differences were not significant. Based on these data, it is suggested that the *-475* and *-631 IL2* polymorphisms as functional promoter position may be involved in *IL2* expression and regulation. To find out the exact effect of the mentioned SNPs on susceptibility to MS, study on a larger sample size is suggested.

## Introduction

Multiple sclerosis (MS) is a chronic neuroinflammatory and autoimmune disease caused as a result of demyelination of the central nervous system. It is thought to be started and mediated by autoreactive T cells directed against myelin antigens. Both genetic and environmental elements are contributed in a significant risk of disease (1).

*interleukin-2 (IL2)* is a kind of cytokine involved in the function and adjustment of immune system. *IL2* is known as pro and anti inflammatory factor. At first, *IL2* was identified as an autocrine secretary product from activated T cells with growth factor characteristics. After some years, it was found that *IL2* could influence the T-cell proliferation, existence and differentiation of effector cells. The *IL2* gene is powerful functional candidate that is involved in immune regulation and operation. The major no redundant role of *IL2* is to maintain peripheral T-cell tolerance; it has a significant role in regulatory T-cell (T-reg) homeostasis. The impairment of T-reg cells is supposed to be the basis of autoimmunity in the nonexistence of *IL2* (2, 3). Many researches point that *IL2* played a significant part in the pathogenesis of MS (4-10). *IL2* gene is situated on chromosome 4q21. Levels of *IL2* increase in their cerebrospinal fluid (CSF) and plasma of MS Patients (11). In a mouse model of empirical autoimmune encephalomyelitis (EAE), *IL2* gene deletion caused a decrease in susceptibility to EAE (11, 12).

Moreover, genetic examination in nonobese diabetic (NOD) mice has determined a locus of lower than 0.15 cm which is significant in both EAE and susceptibility to diabetes; besides, this locus contains the *IL2* gene (13). For the first time, Fedetz et al. assessed *-475* and *-631 IL2* gene polymorphism in Spanish population. They found that these polymorphisms were not significantly more frequent in MS patients than controls (14). The *-330 IL2* polymorphism was evaluated in Iranian and other populations with MS disease. Some of studies indicated that *-330 IL2* position had susceptibility effects, while the others found it as a protective factor (9, 15-19). Meanwhile, the *+114* and *+166 IL2* polymorphisms were evaluated, too. But, there was no significant association between positions and MS disease (9, 15, 18).

In the present study, for the first time, we report the frequency of the *-475* and *-631 IL2* polymorphism positions in Iranian MS patients in comparison with healthy individuals.

## Materials and Methods

### Subjects and control groups

In this case-control study, 100 patients with relapsing remitting MS were included in this work. The age of patient group was 32.95 ± 6.5 with the age range of 20-40 years. They were selected from the medical genetics department of Sarem Women Hospital and examined by a neurologist in accordance with the McDonald criteria (20). One hundred ethnically, sex and age matched healthy individuals with no personal or family background of autoimmune diseases were selected and considered as controls. The controls had mean age of 29 ± 7.8 years and age range of 20-52 years. All subjects were notified of the work and gave their informed consent on paper. The study was approved by a Payame Noor University Local Ethics Committee. Demographic and clinical data of MS and control groups are presented in table 1.

**Table 1:** Demographic and clinical profiles of MS patients and controls


Variables	MS patients	Controls

**Female/Male [No. (%)]**	59 (59%)/41(41%)	60 (60%)/40 (40%)
**Age (mean ± SD, Y)**	32.95 ± 6.51	29.8 ± 7.8
**Age range (Y)**	20-42	20-52
**Age at onset (mean ± SD, Y)**	28.3 ± 4.2	-
**Relapsing-remitting course (No. %)**	100 (100%)	-
**Duration (mean ± SD, Y)**	4.86 ± 5.535	-
**EDSSa (mean ± SD)**	3.775 ± 2.226	-


a; Expanded disability status scale of Kurtzke.

### Genotyping and collection of data

DNA was extracted from peripheral blood samples by employing salting out method (21). For observing the *IL2* single nucleotide polymorphisms (SNPs) at *-475* and *-631* positions, restriction fragment length polymorphism- polymerase chain reaction (RFLP-PCR) was performed in accordance with Fedetz et al. (14). Due to the end result of *-475* and *-631 IL2* genotyping, we managed to define alleles and genotypes which were susceptible. The sequence of primers, length of PCR product, name of restriction enzyme and digested product size were concluded in table 2. For *-475* and *-631 IL2* positions, *Mse* I and *Rsa* I restriction enzymes (New England Biolabs) were used, respectively. The *-475* T allele carriers produced the 248 bp band, while samples having *-475* A allele was digested to the 131 and 117 bp bands. Digestion with RsaI produced a 271 bp band in *-631* G allele carriers. The digestion products were run on 10% polyacrylamide gel

### Statistical analysis

To examine the susceptible *-475* and *-631 IL2* polymorphisms in MS disease, χ^2^ was calculated and Fisher’s exact test was accomplished using SPSS 18v for windows software. P value< 0.05 was regarded as significant.

## Results

In this study, the *-475* and *-631 IL2* genotypes in MS patients and a matching control group were compared using RFLP-PCR. For the *-475* position, T allele was more frequent in the MS patients than controls (2% vs. 0%). The allele comparisons of *-475 IL2* between MS patient and control group were not significant. While 4 patients had A/G genotype, all the controls carried A/A genotype. This difference between the patient and control groups was not significant (p= 0.1, Table 3).

For the *-631* position, the frequency of A allele was low in case and control groups. The findings revealed that A allele and A/G heterozygote genotypes were found in 1% and 2% of MS patients, respectively. Whereas there was no A allele in any of the healthy controls, the difference between MS patients and healthy controls was not significant (p=0.4, Table 4).

**Table 2:** The sequence of primers, length of PCR product, name of restriction enzyme and length of digested product


**Primer sequence**	Forward Primer: 5´- ATAGACATTAAGAGACTTAAAC -3´
	Reverse Primer: 5´-GTAACTCAGAAAATTTTCTTTG -3´
**Length of PCR product**	332 bp
**Name of restriction enzyme**	MseI: for detection of -475 IL2 position
	RsaI: for detection of -631 IL2 position
**Length of digested product**	248 bp: the carriers of -475 T allele
	117 bp and 131 bp: the carriers of -475 A allele
	271 bp: the carriers of -631 G allele


**Table 3:** The frequency of -475 IL-2 gene polymorphism in Iranian MS patients and controls


	Patients (%)	Controls (%)	OR	CI (95%)	P

**Allele**	n=200	n=200	0.980	(0.961-1)	0.123
**A**	196(98%)	200(100%)			
**T**	4(2%)	0			
**Genotype**	n=100	n=100	0.121	(0.922-0.999)	0.121
**A/A**	96(96%)	100(100%)			
**A/T**	4(4%)	0			
**T/T**	0	0


**Table 4:** The frequency of -631 IL-2 gene polymorphism in Iranian MS and control individuals


	Patients (%)	Controls (%)	OR	CI (95%)	P

**Allele**	n=200	n=200	0.990	(0.976-1.004)	0.499
**G**	198(99%)	200(100%)			
**A**	2(1%)	0			
**Genotype**	n=100	n=100	0.980	(0.953-1.008)	0.497
**G/G**	98(98%)	100(100%)			
**G/A**	2(2%)	0			
**A/A**	0	0


## Discussion

The risk variations in 4q27 location which accessed susceptibility to autoimmune diseases, have demonstrated by genomic association study (20-24). Genes such as *IL2* are located in this region which is believed to be strongly associated to autoimmune diseases etiology. Also, the role of anti-inflammatory and antioxidant factors in experimental models of MS (EAE model) in genomic the clinical signs of the disease has been reported (25, 26). As suggested by studies in EAE model and allelic expression, the immune mediated demyelinating process in MS suggested the involvement of *IL2* as a factor of pro-inflammatory and anti-inflammatory in order to promote proliferation of T cell (9). We have already demonstrated an increased plasma concentration of *IL2* in the patients enrolled in this study (12). Therefore, some polymorphisms, especially on promoter region of *IL2* gene may play a major role in pathogenesis of MS disease.

In our study, the frequency of *-475* T allele was higher in MS patients than controls, but this difference was significant as seen on *-631* A allele. These data indicate that *-574* and *-631 IL2* polymorphisms had no significant association with MS disease. Consistent with our study, Fedetz et al. reported that lack of the association between *-475* and *-631 IL2* polymorphisms and MS disease (14). Also, similar to Iranian MS patients, no significant differences were found in the frequency of alleles and genotypes of *-475* A/T and *-631 G/A IL2* in two main Kazakhs and Russians ethnic groups of Kazakhstan (27). Moreover, the frequency of the *-475* T and *-631* A allele are 0.01 and 0.03 in the SNP database, respectively, which is similar to results of our study.

These two SNPs are located in distal region of the *IL2* promoter. Ward et al. reported that this distal region may be served as a stable nucleation and/or a potential initiator site (28). Therefore, the SNPs in this region can be postulated for more research studies. The MS patients had more frequency of *-475* T and *-631* A allele compared to controls (but not significant). Therefore, study on large sample size may discover significant differences in *-475* and *-631 IL2* positions in MS patients.

## Conclusion

Frequency of both polymorphisms (*-475* and *-631 IL2* positions) were slightly greater in MS patients in comparison with controls, but these frequency differences were not significant. Since *-475* and *-631 IL2* polymorphisms had been suggested as a functional promoter position, study on large sample size are required to bring about more authentic results.
